# Supplementary material for: Functions of 1,25-dihydroxy vitamin D3, vitamin D3 receptor and interleukin-22 involved in pathogenesis of gout arthritis through altering metabolic pattern and inflammatory responses
Source: PeerJ. 2021 Dec 1;9:e12585. doi: 10.7717/peerj.12585 (PMC8643101; doi:10.7717/peerj.12585)
Supplement: Supplemental Information 1 [file peerj-09-12585-s001.zip › rawa/GO,METABOLOMIC/network.zip!/network/string_interactions.tsv.pdf]

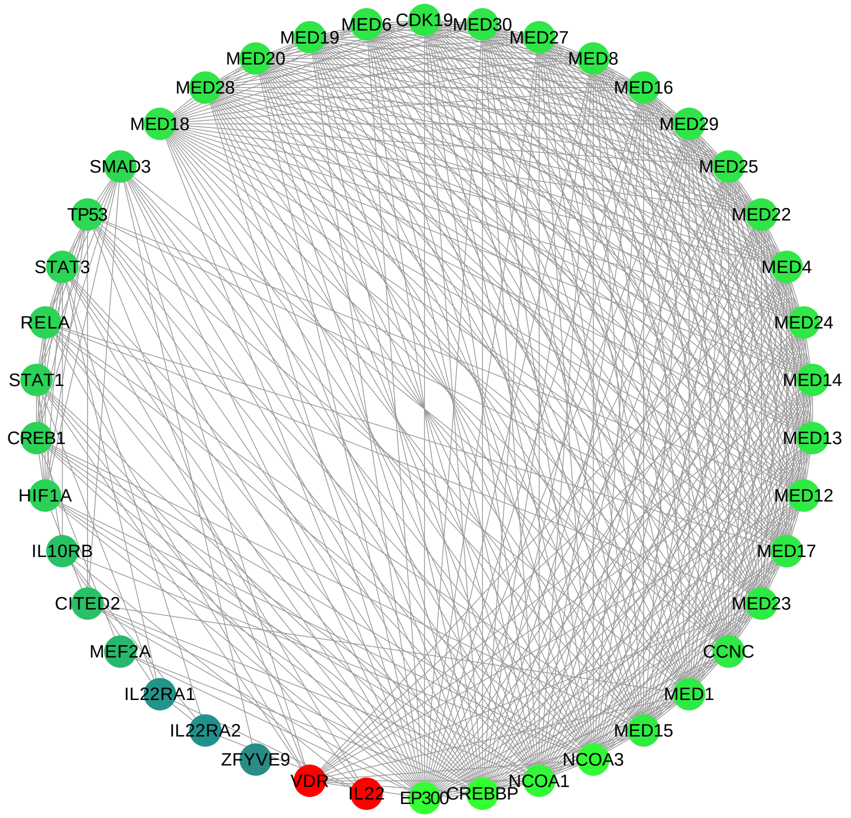
CDK19
MED30
MED27
MED8
MED16
MED29
MED25
MED22
MED4
MED24
MED14
MED13
MED12
MED17
MED23
CCNC
MED1
MED15
NCOA3
NCOA1
CREBBP
EP300
IL22
VDR
ZFYVE9
IL22RA2
IL22RA1
MEF2A
CITED2
IL10RB
HIF1A
CREB1
STAT1
RELA
STAT3
TP53
SMAD3
MED18
MED28
MED20
MED19
MED6
